# Socio-emotional and adaptive behaviour in children treated for severe anaemia at Lira Regional Referral Hospital, Uganda: a prospective cohort study

**DOI:** 10.1186/s13034-020-00352-4

**Published:** 2020-11-26

**Authors:** Andrew Sentoogo Ssemata, Robert Opika Opoka, John Mbaziira Ssenkusu, Noeline Nakasujja, Chandy C. John, Paul Bangirana

**Affiliations:** 1 Department of Psychiatry, Makerere University, College of Health Sciences, P.O. Box 7072, Kampala, Uganda; 2 Department of Paediatrics and Child Health, Makerere University, College of Health Sciences, Kampala, Uganda; 3 Department of Epidemiology and Biostatistics, Makerere University, College of Health Sciences, Kampala, Uganda; 4 Ryan White Center for Pediatric Infectious Disease & Global Health, School of Medicine, Indiana University, Indianapolis, IN USA

**Keywords:** Neurodevelopment, Pre-school, Children, Severe anaemia, Adaptive behaviour, Socio-emotional, Uganda

## Abstract

**Background:**

Severe anaemia is a global public health challenge commonly associated with morbidity and mortality among children < 5 years of age in Sub-Saharan Africa. However, less is known about the behavioural performance of children < 5 years surviving severe anaemia in low resource settings. We investigated social-emotional and adaptive behaviour in children < 5 years diagnosed with severe anaemia in Northern Uganda.

**Methods:**

We conducted a hospital based prospective cohort study among children 6—42 months who were treated for severe anaemia (n = 171) at Lira Regional Referral Hospital, Uganda. Socio-emotional and adaptive behaviour were assessed 14 days post discharge using the Bayley Scales of Infant and Toddler Development, 3rd edition. Age-adjusted z-scores for each domain were calculated using scores from healthy community children (n = 88) from the same environment for each age category. Multiple linear regression was used to compare z-scores in the social-emotional and adaptive behaviour scales between the two groups after adjusting for weight-for-age z-score, social economic status, mother’s education, father’s education and father’s employment on all the scales.

**Results:**

Compared with healthy community controls, children with severe anaemia had poorer [adjusted mean scores (standard error)], socio-emotional [− 0.29, (0.05) vs. 0.01, (0.08), P = 0.002]; but not overall/ composite adaptive behaviour [− 0.10, (0.05) vs. − 0.01, (0.07), P = 0.343]. Within the adaptive behaviour subscales, children with SA displayed significantly poorer scores on the community use [adjusted mean score (standard error)], [− 0.63, (0.10) vs. − 0.01, (0.13), P < 0.001]; and leisure [− 0.35, (0.07) vs. − 0.02, (0.07), P = 0.036] skills.

**Conclusion:**

This study suggests that severe anaemia in children < 5 years is associated with poor social-emotional scores in the short-term post clinical recovery in Northern Uganda. We recommend long-term follow-up to determine the course of these problems and appropriate interventions to reduce the behavioural burden among children < 5 years surviving severe anaemia in Uganda.

## Background

Severe anaemia (SA) defined as haemoglobin (Hb) < 5 g/dL, is a common public health problem among children under 5 years of age in resource-constrained areas with the highest mean severity in all low and middle income regions [1]. The prevalence of SA among African children is notably high with a burden close to 63% and significant in-hospital paediatric morbidity and mortality [2]. In malaria endemic areas, severe anaemia is a prevalent complication of malaria among African children especially those below 5 years in endemic countries [3, 4].

Severe anaemia (Hb < 5 g/dL) is associated with frequent infections, haemoglobinopathies, micronutrient deficiencies and inadequate feeding practices causing life-long health consequences including diffused cerebral hypoxia, tissue ischaemia, cognitive dysfunction, impaired cerebral vascular regulation, neurological injury and mortality [5–10]. Evidence that the brain is vulnerable during acute and chronic anaemia (Hb < 7 g/dL) is provided by human and animal studies where acute anaemia has been linked to cognitive dysfunction and evidence of cerebral cellular hypoxia [11–13]. Severe anaemia may lead to brain dysfunction and cerebral injury with the brain white matter identified as the predominant site of injury [13–15].

Severe anaemia-induced injury may affect child growth and development including difficulties in learning complex task, short term memory deficits and decreased motor control [13, 15]. The behavioural effects are of greatest concern because they can persist beyond treatment and resolution of anaemia [7, 16]. They may affect the mental, physical and social growth and development in children affecting their academic and career prospects later in life [7, 9, 17].

Majority of research has focussed on various forms of anaemia and cognitive outcomes [15, 18, 19]. The few available studies generally show that children in a low-income community with mild anaemia defined as Hb < 11 g/dL display less social looking toward their mothers, are slower to display positive affect and display greater wariness and hesitancy to touch novel toys for the first time compared to their non-anaemic counterparts [20]. Similarly, a recent study among 252 Ugandan children < 5 years surviving severe malaria showed that severe malarial anaemia (SMA) was associated with internalizing (emotional wellbeing i.e. somatic complaints without know cause, anxiety, depression, emotionally reactive, withdrawal) and externalizing (behaviour toward others i.e. attention problems, aggressive behaviour) and total behavioural problems at 12 and 24 months [21]. In this study, a behavioural assessment using the Child behaviour checklist (CBCL) was conducted for children who had severe malarial anaemia.

Behavioural outcomes after severe anaemia (Hb < 5 g/dL) remain largely unknown and receive little or no attention in many public health spheres; despite SA causing so much injury and disability [14, 15]. This is partly due to the fact that emphasis is usually focussed on SA resolution and the reduction of risk factors of SA; yet the effect of anaemia on a child’s behaviour may contribute to poorer cognitive outcomes [3, 4]. We focus on severe anaemia as previous research has shown that clinical severity of symptoms during the acute phase of illness has significant effect on a wider variability of functional areas and performance outcomes [22, 23]. To address the gaps in knowledge regarding behavioural outcomes in children with SA, we conducted a prospective cohort study using the Bayley Scales of Infant and Toddler Development, 3rd Edition (Bayley–III) [24, 25] to characterize the effect of SA on socio-emotional and adaptive behaviour among Ugandan children below 5 years in the immediate period post recovery. These preschool years have been reported as a time of great cognitive, psychological and behavioural growth and brain development involving dynamic and elaborative developmental changes [26]. Therefore, examining this effect during formative early years among children in a low resource setting who are at greatest risk of SA and behavioural development disruption may be clinically important for public health and may direct early intervention services. We hypothesized that children with SA would have poorer socio-emotional and adaptive behavioural scores compared to community children.

## Methods

This was a prospective study with a sample of 259 children (171 with severe anaemia and 88 community controls) aged 6–42 months conducted between August 2016 and June 2017 at Lira Regional Referral Hospital in Northern Uganda. Participants with SA were in-patients of an implementation research study on management and outcomes of severe anaemia in Ugandan children where SA was defined as Hb < 5 g/dL [27, 28].

Parents of children with SA were informed about the need for healthy control children in the study age range to generate a control group. The non-febrile healthy community children (CC) were siblings from their nuclear or extended family, within the household compound area or neighbours (playmate) residing in the same locality (< 1 km) of the enrolled children with SA who had been volunteered by the parents after invitation to participate in the study. The utilization of siblings or neighbours was to minimize differences between groups in relation to family background, household environment, socio-environment/ neighbourhood, culture and linguistics, number of siblings in the household, number of meals per day.

The CC were aged between 6 and 42 months, and healthy. They were examined at the time of enrolment to ensure that they did not have clinical pallor on clinical examination of the palms, nail-beds, tongue and conjunctivae (as a method of SA assessment) or a history of hospitalization for SA 6 months prior to enrolment. Assessment of SA using clinical signs such as pallor is a commonly used method (especially in our settings with poor or absent diagnostic facilities) for diagnosis of severe anaemia in children with sensitivity and specificity ranging from 53 to 96% [23, 29]. The children in the SA and CC groups were otherwise identical and sampled at the same time to minimise any biases.

### Clinical and demographic assessment

Social economic status (SES) and demographic characteristics were obtained using a questionnaire of material possessions assessing housing quality, cooking resources, water accessibility and the presence of key amenities (radio, shoes for subject, mobile phone, poultry) in which lower SES scores have been associated with worse cognitive functioning in healthy Ugandan paediatric population under 5 years of age [30]. Nutritional status was obtained by comparing anthropometric indicators (weight-for-age (WAZ), height for age (HAZ) and weight for height (WHZ) using the WHO anthro survey analyser software for children under 5 years of age to generate the standardized z-scores [31]. We followed internationally recognised cut-offs to consider children whose HAZ, WAZ, or WHZ fall more than two SDs below the international mean to be stunted, underweight or wasted, respectively [32].

### Behavioural assessment

Behavioural assessment was done using the Bayley Scales of Infant and Toddler Development, 3rd edition (Bayley-III). It is one of the most commonly adapted comprehensive psychometric assessment tools used in research, in clinical practice, and to evaluate interventions as it assesses several developmental domains as a measure of early global development among very young children [24, 25, 33]. The interviews with the caregivers were conducted in a quiet room with minimal distractions at the hospital. For uniformity and language concerns, trained assessors with Bachelor’s degrees in Psychology and fluent in Langi (the local dialect of the study setting) administered the test to the child’s primary caregiver. The first author (Health psychologist) supervised the administration of interview questionnaire and reviewed the record forms at the end of each week for completeness to ensure data quality. The assessors were periodically given review trainings by a psychology graduate independent of the study and with expertise in Bayley-III. For consistency and to ensure data quality, the team (two interviewers and first author) met regularly to review, feedback and discuss the data.

Assessments were conducted 14 days post discharge for the caregivers of the children with SA and at enrolment for the CCs or when appropriate for the caregiver to return to the hospital for assessment. We interviewed the primary caregiver of each child using the social–emotional and adaptive behaviour scales of the Bayley–III [24, 25]. Bayley-III has not been validated for our setting however has been adapted for appropriate use among Ugandan children in previous studies and used for rural populations similar to our study sample and setting [34–36].

Majority of the caregivers were mothers, familiar with the child and could provide meaningful, accurate and complete response ratings of their child’s personal, adaptive and social skills necessary for daily living. The social-emotional scale assesses emotional and social development as well as sensory processing that influences a child’s emotional responses based on the Greenspan Social-Emotional Growth Chart [37]. The scale provides a general indication of a child’s level of social-emotional development and presence or absence of sensory processing difficulties [38]. The scale assesses the child’s functional, social and emotional milestones namely; self-regulation and interest in the world, relationship engagement, emotional engagement in an interactive and purposeful manner, communication with interactive emotional gestures, problem solving through interactive emotional gestures, communicating intentions and feelings using symbols and ideas, using symbols to express intentions, wishes or feelings more than basic needs, creating logical bridges between ideas and emotions [24, 37, 38].

Adaptive behaviour is a collection of skills (conceptual, social, and practical) for effective functioning that concern the way individuals meet their personal needs while meeting their demands in their environment [39, 40]. The adaptive behaviour scale is derived from items for children 0–5 years of the Parent/Primary Caregiver Form of the Adaptive Behaviour Assessment Scale–Second Edition-ABAS-II [41]. The scale assesses ten areas categorized in three broader domains: [1] conceptual (communication, functional academics, and self-direction); [2] social (social and leisure); and [3] practical (self-care, home or school living, community use, health and safety) [39, 42]. A summation of the ten sub-scales composite scores was obtained to generate an overall adaptive behaviour score also known as the General Adaptive Composite (GAC) score.

### Statistical methods

Data were entered into Filemaker 11.0v3 (FileMaker Inc. US) database, and exported into IBM SPSS 23 (IBM Corp., Armonk, N.Y., USA) for statistical analysis. For this study, raw scores for each scale were converted into an age and sex-specific standardized z-score, based on the scores of healthy community children (CC, n = 88). The z-scores were computed as (actual score–mean score for a child’s sex and age)/SD, where the mean score for a child’s sex and age and SD were computed by fitting a linear regression model to data for all CC children [18]. Z-scores have a mean of 0 and SD 1 in the CC reference population. Multiple linear regression was used to compare z-scores on all the scales between the two groups after adjusting for weight-for-age z-score, social economic status, mother’s education, father’s education and father’s employment. We adjusted for multiple testing for the adaptive subscales using the Hommel’s procedure [43] and *P* < 0.05 was statistically significant.

### Ethics

Approvals for this study were obtained from Makerere University School of Medicine Research Ethics Committee (REC Ref: 2015–045), Uganda National Council for Science and Technology (Ref: HS 2017) and the Lira Regional Referral Hospital administration. Participation in the study was voluntary and the caregivers of the study participants who took part in the study provided written informed consent.

## Results

### Characterization of the study sample children

We interviewed caregivers of 259 children (171 with SA and 88 CCs) with overall mean age of 1.94 years (CCs, M = 2.07 years, SD = 0.96; SA, M = 1.88 years, SD = 0.94). The children in this study resided in the same geographical region and their characteristics are described in Table 1. Children with SA had lower social economic status, mother’s education, father’s education and father’s employment statuses than CC children.

**Table 1:** Demographic characteristics of severe anaemia and control study children

Characteristic	Severe anaemia (n = 171)	Community children (n = 88)	P value
Age in years, mean (SD)	1.88 (0.94)	2.07 (0.96)	0.129
Female sex, n (%)	75 (43.9)	47 (53.4)	0.145
Nutritional status indicators^a^			
Underweight (WAZ < -2 SD) n (%)	10(6.2)	13 (14.9)	0.023*
Stunting (HAZ < -2 SD) n (%)	15(11.9)	10 (11.8)	0.975
Wasting (WHZ < -2 SD) n (%)	11(8.3)	12 (14.0)	0.284
Social economic status wealth indices, n (%)			0.048*
Poor	44 (25.7)	10 (11.4)	
Second	30 (17.5)	21 (23.9)	
Middle class	31 (18.1)	24 (27.3)	
Fourth	36 (21.1)	20 (22.7)	
Wealthy	30 (17.5)	13 (14.8)	
Maternal education level, n (%)			0.038*
Primary or less	151 (88.3)	69 (78.4)	
Secondary	15 (8.7)	14 (16.0)	
Tertiary	5 (3.0)	5 (5.6)	
Paternal education level, n (%)			0.036*
Primary or less	120 (70.2)	48 (54.5)	
Secondary	26 (15.2)	14 (16)	
Tertiary	25 (14.6)	26 (29.5)	
Maternal employment n (%)			0.153
Yes	20 (11.7)	16 (18.2)	
No	151 (88.3)	72 (81.8)	
Paternal employment n (%)			0.039*
Yes	74 (43.3)	50 (56.8)	
No	97 (56.7)	38 (43.2)	

^a^The number of children assessed differed from the total number of children WAZ (n = 249); HAZ (n = 211); WHZ (n = 218)

^*^P ≤ 0.05 is statistically significant

### Behavioural performance

Compared with healthy community controls, children with SA displayed significantly poorer scores on the socio-emotional [adjusted mean score (SE)], [− 0.29, (0.05) vs. 0.01, (0.08), *P* = 0.002] but not the composite adaptive behaviour [− 0.10, (0.05) vs. − 0.01, (0.07) *P* = 0.343] scales (Table 2). We also compared the SA group against the CCs group on the adaptive behaviour subscales. We found that children with SA displayed significantly poorer scores on the community use [adjusted mean score (standard error)], [− 0.63, (0.10) vs. − 0.01, (0.13), P < 0.001]; and leisure [− 0.35, (0.07) vs. − 0.02, (0.07), P = 0.036] skills subscales. Compared to the CC group, children with SA had lower scores on the communication, functional pre-academics, self-direction, home living and health and safety, self-care, social and motor scales but these were not statistically significant (Table 2).

**Table 2:** Adjusted means for social-emotional and adaptive behavioural z-scores for children with severe anaemia and community control children

Scales	Severe Anaemia n = 171 M, (SE)	Community Control n = 88 M (SE)	Difference in means p-value
Social emotional	− 0.29, (0.05)	0.01, (0.08)	0.002*
Overall Adaptive behaviour	− 0.10, (0.05)	− 0.01, (0.07)	0.343
Adaptive communication	− 0.11, (0.05)	0.00, (0.07)	0.776
Adaptive community use^a^	− 0.63, (0.10)	− 0.01, (0.13)	< 0.001*
Adaptive pre-academics^a^	0.06, (0.12)	− 0.02, (0.16)	0.976
Adaptive home living^a^	0.26, (0.12)	− 0.03, (0.16)	0.656
Adaptive health and safety	− 0.11, (0.05)	− 0.01, (0.07)	0.931
Adaptive leisure	− 0.35, (0.07)	− 0.02, (0.09)	0.036*
Adaptive self-care	− 0.26, (0.06)	− 0.03, (0.09)	0.224
Adaptive self-direction	0.03, (0.08)	− 0.03, (0.11)	0.976
Adaptive social	0.25, (0.08)	− 0.04, (0.11)	0.273
Adaptive motor	0.00, (0.06)	0.00, (0.08)	0.976

^a^Children below one year are not assessed on these skill areas and the total number of children for these scales for each group were–Severe anaemia group n = 126; Control group n = 70)

Associations with adaptive subscales have been adjusted for multiple testing using the Hommel’s procedure (Hommel [43]) and *p < 0.05 is statistically significant

## Discussion

This study set out to examine the effect of SA on social-emotional and adaptive behaviour using the Bayley-III among Ugandan children aged 6–42 months in the immediate period post recovery in Lira district, Northern Uganda. The study findings showed that SA is associated with poor social-emotional behaviour among Ugandan children < 5 years of age. We found no significant differences between the two groups on overall adaptive behaviour. These results reflect the potential effect of severe anaemia that greatly affects African children < 5 years in resource-limited setting [2]. One possible explanation for these results is that the altered social-emotional behaviour may be accounted for by the alterations in the frontal-striatal circuits and the mesolimbic/meso-cortical dopamine levels as observed among children with iron deficiency anaemia [44]. Though not assessed in the present study, iron deficiency is estimated to be the commonest contributor to the aetiology of severe anaemia and these iron status changes affects certain brain regions [45]. The dysfunction could be due to direct effect of low haemoglobin on neurodevelopment or the systemic inflammation caused by anaemia leading to failure to provide oxygen to the brain tissues in children [44]. These alterations in social-emotional behaviour are associated with poor overall developmental outcomes and may affect school performance, personal relationships and consequently adaptive behaviour that draws together a person’s cognitive and personality characteristics [39, 46]. Consequently, children with SA will be poor at negotiating complex social-emotional patterns and reaching functional emotional milestones that provide purpose to mental processes [24].

It is important to note that altered social-emotional behaviour as observed in the present study has been reported to affect how children react and experience their social and physical environment; thereby fuelling poor developmental outcomes that could significantly affect children’s growth and development [47]. Children’s social-emotional development is reported to enhance children’s adaptive behaviour, safety, home living, health, social relationships, self-awareness, emotional regulation, independence, academic outcomes and lifelong learning [48, 49]. This may be a possible explanation for the significantly poor performance on community use and leisure skills among children with SA. Community use and leisure skills have been reported to hamper skills needed to function in the community for example adequately exploring the environment, home living (helping with chores), health and safety (following safety rules and avoiding physical danger), and self-care (eating, dressing, toileting, brushing teeth) among infants and pre-schoolers [46]. This could indicate that children with SA may not achieve their potential across multiple adaptive behaviour skills particularly in the social (leisure) and practical (community use) domains; which are necessary for young children to become increasingly more independent. Deficits in these domains during the critical child development period, have the potential to affect the pre-schoolers’ key functional developmental tasks [39, 50]. As a result, children with SA may be unable to encounter daily needs and manage the natural and social demands of the environment critical to child survival.

Furthermore, adaptive behaviours are intricately connected to other developmental domains such as cognition, motor and language skills; and as children grow older and begin to exhibit more sophisticated behaviour [39–41, 51]. Therefore, SA has the propensity to compromise brain–behaviour development and lead to slower developmental trajectory. This further creates difficulties in cognitive processing and school achievement which have a great bearing on the child’s health, well-being, and quality of life [15, 20, 44]. The socio-emotional and adaptive behaviour problems post SA in children < 5 years could potentially create a heightened risk for the impaired neurocognitive outcomes. Therefore, understanding the adaptive skills affected by SA in children may support the tailoring of interventions aimed at improving their functional outcomes. These interventions should focus on assessing and understanding the socio-emotional, sensory processing and adaptive skills present in early childhood [39]. This is essential as these domains reflect the needs and feelings during the early years of life; critical to a child’s future and survival [39, 41].

While research on neurodevelopmental impairment among children < 5 years surviving SA in LMICs is limited, this study indicates that children with SA have poorer social-emotional behaviour compared to their healthy counterparts. Understanding the social, emotional and behavioural development of children after illness is important as it reflects the critical aspects of the child’s well-being, awareness of risks to brain function and the physiological adaptation to disease or environmental influences on brain development during formative early years [33]. We advocate for screening and early identification of neurobehavioral deficits among children with SA post-discharge and linkage to rehabilitations services in resource-constrained settings to avert further developmental challenges.

Therefore, additional research on the neurodevelopmental needs of children with SA and the integration of early childhood development services into paediatric SA treatment programmes in LMICs is recommended. Assessing adaptive behaviour and functional abilities focuses on an essential dimension of human function essential in the diagnosis of impairment and intellectual disability [40].

### Strength and limitations

Our study provides important and novel data on the effect of SA on the socio-emotional behaviour among Ugandan children in a resource-constrained setting. Realizing the level and risk of disruption of brain development during formative early years due to SA and identifying the neurodevelopmental concerns early in life may direct early intervention services aimed at averting the impairment trajectories and improve functionality. The study also highlights the need for early and appropriate interventions targeted towards the earliest years across all skill areas such as computerised cognitive rehabilitation therapy (CCRT) [52] and Mediational Intervention for Sensitizing Caregivers (MISC) [53] to avert socio-emotional and behavioural challenges these children may develop. This will reduce the burden of future developmental risk and dysfunction that may be associated with severe anaemia among children in Uganda and other resource-constrained countries in Sub-Saharan Africa that could further burden the health system.

The current study has limitations worth noting. The aetiology of SA was largely symptomatic. We were not able to collect any further clinical data like malaria diagnosis as the study area is a region of high malaria transmission intensity. However, it is worth noting that anaemia among children in LMICs is a result of micronutrient deficiencies, acute and chronic inflammation, malnutrition and frequent infections [10] that may also affect socio-emotional behaviour. This study did not assess post transfusion Hb or if SA had cleared prior to assessment as both anaemia and transfusion are independently associated with organ injury and increased morbidity [5]. Future research should assess post-discharge Hb prior to assessment. Socio-emotional and adaptive behaviour were assessed by caregiver reports of aspects of their child’s development basing on their perceptions of their children and may be over- or under-represented. Parental reporting may be less valid in settings where the background level of awareness about early child development is low guided by cultural values and norms with a bias to portray their child in a positive light [48].

This was a prospective cohort study assessing the socio-emotional and adaptive behavioural skills performance in a resource-constrained setting at a single time point therefore causality could not be established and findings may not be representative of the entire population. However we used healthy community children as a comparator group for normal test results in this age group in this area.

Our study has critical implications for the design and delivery of interventions, among children especially those below 5 years in low resource malaria endemic settings where the burden of SA is high among children under 5 years who are at greater risk of severe anaemia acquisition. Severe anaemia in early childhood is one of the key conditions that affects the brain associated with dysfunction and injury [15, 54]. Therefore as reflected by our study findings SA may contribute to significant behavioural impairment. As more children are treated and survive SA, these children go on to grow with consequent socio-emotional and adaptive behavioural problems. Given the effects of SA on socio-emotional and adaptive behavioural outcomes, adoption of effective interventions such as CCRT and MISC [52, 53] that have been used to enhance neurodevelopment among African children with conditions such as malaria and HIV are very much needed. Similar interventions could be adopted for children with SA to enhance neurobehavioral function.

Our study findings are clinically important for health care providers, researchers, caregivers and policy makers. Considering the heightened risk of infections such as malaria, HIV and subsequent SA in the formative years of brain development, additional strategies to prevent and treat anaemia before it progresses to severe staging are urgently needed to reduce neurobehavioral deficits that may result from poor disease management among children in environments with high SA incidence [23].

## Conclusion

The investigations of this study demonstrate that severe anaemia is associated with poorer social-emotional behaviour among children aged 6 to 42 months in Uganda. Deficits reported in these areas could become significant risk factors for the later development of social and academic difficulties. The evaluation of socio-emotional and adaptive behaviour among children surviving SA may have important diagnostic, clinical, and therapeutic implications more so as these assessments are not part of standard of care in many LMICs. Additionally, the findings affirm the rationale for prompt treatment and management of anaemia before it progresses to severe staging in order to contain the neurobehavioral deficits in these children. Future research is needed to evaluate the long-term course of these behavioural problems, their risk factors and to develop targeted interventions.

## Data Availability

All data generated or analysed during this study supporting the conclusions of this article are included in this manuscript text [and its tables].

## Abbreviations

Bayley-III: Bayley Scales of infant and toddler development, 3rd edition
CC: Community children
GAC: General adaptive composite
HAZ: Height-for-age z-score
Hb: Haemoglobin
SA: Severe anaemia
SES: Social economic status
SMA: Severe malarial anaemia
WAZ: Weight-for-age z-score
WHZ: Weight-for-height z-score

## References

[1] Kassebaum NJ, Jasrasaria R, Naghavi M, Wulf SK, Johns N, Lozano R (2014). A systematic analysis of global anemia burden from 1990 to 2010. Blood.

[2] Stevens GA, Finucane MM, De-Regil LM, Paciorek CJ, Flaxman SR, Branca F (2013). Global, regional, and national trends in haemoglobin concentration and prevalence of total and severe anaemia in children and pregnant and non-pregnant women for 1995–2011: a systematic analysis of population-representative data. Lancet Global Health.

[3] Biemba G, Dolmans D, Thuma PE, Weiss G, Gordeuk VR (2000). Severe anaemia in Zambian children with Plasmodium falciparum malaria. Tropical Med Int Health.

[4] Crawley J (2004). Reducing the burden of anemia in infants and young children in malaria-endemic countries of Africa: from evidence to action. Am J Tropic Med Hygiene..

[5] Shander A, Javidroozi M, Ozawa S, Hare G (2011). What is really dangerous: anaemia or transfusion?. Br J Anaesthesia..

[6] Casals-Pascual C, Idro R, Gicheru N, Gwer S, Kitsao B, Gitau E (2008). High levels of erythropoietin are associated with protection against neurological sequelae in African children with cerebral malaria. Proc Natl Acad Sci.

[7] Balarajan Y, Ramakrishnan U, Özaltin E, Shankar AH, Subramanian S (2012). Anaemia in low-income and middle-income countries. The Lancet.

[8] McCann JC, Ames BN (2007). An overview of evidence for a causal relation between iron deficiency during development and deficits in cognitive or behavioral function–. Am J Clin Nutrit.

[9] World Health Organization (2015). The global prevalence of anaemia in 2011.

[10] Perkins DJ, Were T, Davenport GC, Kempaiah P, Hittner JB, Ong'echa JM (2011). Severe malarial anemia: innate immunity and pathogenesis. Int J Biol Sci.

[11] Li M, Bertout JA, Ratcliffe SJ, Eckenhoff MF, Simon MC, Floyd TF (2010). Acute anemia elicits cognitive dysfunction and evidence of cerebral cellular hypoxia in older rats with systemic hypertension. Anesthesiol J Am Soc Anesthesiol..

[12] El Hasnaoui-Saadani R, Pichon A, Marchant D, Olivier P, Launay T, Quidu P (2009). Cerebral adaptations to chronic anemia in a model of erythropoietin-deficient mice exposed to hypoxia. Am J Physiol Regulat Integrat Comparat Physiol.

[13] Weiskopf RB, Kramer JH, Viele M, Neumann M, Feiner JR, Watson JJ (2000). Acute severe isovolemic anemia impairs cognitive function and memory in humans. Anesthesiol J Am Soc Anesthesiol..

[14] Loureiro B, Martinez-Biarge M, Foti F, Papadaki M, Cowan FM, Wusthoff CJ (2017). MRI patterns of brain injury and neurodevelopmental outcomes in neonates with severe anaemia at birth. Early Human Dev.

[15] Hare GM, Tsui AK, McLaren AT, Ragoonanan TE, Yu J, Mazer CD (2008). Anemia and cerebral outcomes: many questions, fewer answers. Anesth Analg.

[16] World Health Organization. The global prevalence of anaemia in 2011. 2015.

[17] Clarke SE, Jukes MC, Njagi JK, Khasakhala L, Cundill B, Otido J (2008). Effect of intermittent preventive treatment of malaria on health and education in schoolchildren: a cluster-randomised, double-blind, placebo-controlled trial. The Lancet.

[18] Bangirana P, Opoka RO, Boivin MJ, Idro R, Hodges JS, Romero RA (2014). Severe malarial anemia is associated with long-term neurocognitive impairment. Clin Infect Dis.

[19] Shaw JG, Friedman JF (2011). Iron deficiency anemia: focus on infectious diseases in lesser developed countries. Anemia..

[20] Lozoff B, Corapci F, Burden MJ, Kaciroti N, Angulo-Barroso R, Sazawal S (2007). Preschool-aged children with iron deficiency anemia show altered affect and behavior. J Nutrit.

[21] Ssenkusu JM, Hodges JS, Opoka RO, Idro R, Shapiro E, John CC (2016). Long-term behavioral problems in children with severe malaria. Pediatrics.

[22] Holding P, Boivin MJ (2013). The assessment of neuropsychological outcomes in pediatric severe malaria. Neuropsychology of children in Africa.

[23] Phiri KS, Calis JC, Faragher B, Nkhoma E, Ng'oma K, Mangochi B (2008). Long term outcome of severe anaemia in Malawian children. PLoS ONE.

[24] Bayley N (2006). Bayley scales of infant and toddler development: technical manual San Antonio.

[25] Bayley N (2006). Bayley scales of infant and toddler development Third Edition.

[26] Brown TT, Jernigan TL (2012). Brain development during the preschool years. Neuropsychol Rev.

[27] Opoka OR, Ssemata SA, Oyang W, Nambuya H, John CC, Tumwine KJ, et al. The management of severe anaemia in children admitted to Ugandan hospitals: high rate of unnecessary blood transfusions due to inadequate utilisation of laboratory services. 2018. 10.1186/s12913-018-3382-5 PMC6052584 30021576

[28] Opoka RO, Ssemata AS, Oyang W, Nambuya H, John CC, Karamagi C (2019). Adherence to clinical guidelines is associated with reduced inpatient mortality among children with severe anemia in Ugandan hospitals. PLoS ONE.

[29] van Hensbroek MB, Jonker F, Bates I (2011). Severe acquired anaemia in Africa: new concepts. Br J Haematol.

[30] Bangirana P, John CC, Idro R, Opoka RO, Byarugaba J, Jurek AM (2009). Socioeconomic predictors of cognition in Ugandan children: implications for community interventions. PLoS ONE.

[31] World Health Organization (2006). WHO child growth standards: length/height-for-age, weight-for-age, weight-for-length, weight-for-height and body mass index-for-age. Methods and development WHO (nonserial publication).

[32] De Onis M, Blössner M (2003). The World Health Organization global database on child growth and malnutrition: methodology and applications. Int J Epidemiol.

[33] Kammerer B, Isquith PK, Lundy S, Boivin MJ, Bruno G (2013). Approaches to assessment of very young children in Africa in the context of HIV. Neuropsychology of children in Africa.

[34] Muhoozi GK, Atukunda P, Diep LM, Mwadime R, Kaaya AN, Skaare AB (2018). Nutrition, hygiene, and stimulation education to improve growth, cognitive, language, and motor development among infants in Uganda: a cluster-randomized trial. Mater Child Nutrit.

[35] Muhoozi GK, Atukunda P, Mwadime R, Iversen PO, Westerberg AC (2016). Nutritional and developmental status among 6-to 8-month-old children in southwestern Uganda: a cross-sectional study. Food Nutrit Res.

[36] Singla DR, Kumbakumba E, Aboud FE (2015). Effects of a parenting intervention to address maternal psychological wellbeing and child development and growth in rural Uganda: a community-based, cluster-randomised trial. Lancet Global Health.

[37] Greenspan SI (2004). Greenspan social-emotional growth chart: a screening questionnaire for infants and young children.

[38] Breinbauer C, Mancil LT, Greenspan SI, Weiss LG, Oakland T, Aylward GP (2010). The Bayley-III Social-Emotional Scale. Bayley-III clinical use and interpretation.

[39] Harman LJ, Smith-Bonahue MT, Weiss GL, Oakland T, Alylward G (2010). The Bayley-III Adaptive Behavior Scale. Bayley-III Clinical use and interpretation.

[40] Tassé MJ, Schalock RL, Balboni G, Bersani H, Borthwick-Duffy SA, Spreat S (2012). The construct of adaptive behavior: Its conceptualization, measurement, and use in the field of intellectual disability. Am J Intell Develop Disabilit.

[41] Harrison PL, Oakland T (2003). Adaptive behavior assessment system- Second Edition (ABAS-II).

[42] Albers CA, Grieve AJ (2007). Review of bayley scales of infant and toddler development. J Psychoeducat Assess.

[43] Hommel G (1988). A stagewise rejective multiple test procedure based on a modified Bonferroni test. Biometrika.

[44] Lozoff B, Clark KM, Jing Y, Armony-Sivan R, Angelilli ML, Jacobson SW (2008). Dose-response relationships between iron deficiency with or without anemia and infant social-emotional behavior. J Pediatr..

[45] Boele van Hensbroek M, Calis JC, Phiri KS, Vet R, Munthali F, Kraaijenhagen R (2010). Pathophysiological mechanisms of severe anaemia in Malawian children. PLoS ONE.

[46] Kirchner RM, Martens MA, Andridge RR (2016). Adaptive behavior and development of infants and toddlers with Williams syndrome. Front Psychol.

[47] Lozoff B, Castillo M, Clark KM, Smith JB, Sturza J (2014). Iron supplementation in infancy contributes to more adaptive behavior at 10 years of age. J Nutrit.

[48] Ren L, Garcia AS, Esteraich JM, Encinger A, Raikes HH, Acar IH (2019). Parent-child relationships and preschoolers' social-emotional functioning among low-income families: the moderating role of parental nativity. Infants Young Children.

[49] Trawick-Smith J (2013). Early childhood development: A multicultural perspective.

[50] Schalock RL, Borthwick-Duffy SA, Bradley VJ, Buntinx WH, Coulter DL, Craig EM, et al. Intellectual disability: Definition, classification, and systems of supports: ERIC; 2010.

[51] Albers CA, Grieve AJ (2007). Test review: Bayley, N. (2006) Bayley scales of infant and toddler development–third edition San Antonio TX Harcourt assessment. J Psychoeducat Assess..

[52] Bangirana P, Boivin MJ, Giordani B (2013). Computerized cognitive rehabilitation therapy (CCRT) for African Children: Evidence for neuropsychological benefit and future directions. Neuropsychology of Children in Africa.

[53] Klein PS, Shohet C, Givon D (2017). A Mediational Intervention for Sensitizing Caregivers (MISC): a cross-cultural early intervention. Handbook of Applied Developmental Science in Sub-Saharan Africa.

[54] Hare GM (2004). Anaemia and the brain. Curr Opin Anesthesiol.

